# Occurrence and Impact of Intraoperative Anastomotic Leakage in Retzius-Sparing Robot-Assisted Radical Prostatectomy

**DOI:** 10.3390/medicina61050886

**Published:** 2025-05-13

**Authors:** Jian-Kai Chen, Yu-Jun Chang, Chi-Bo Lin, Yueh Pan, Pai-Fu Wang

**Affiliations:** 1 Divisions of Urology, Department of Surgery, Changhua Christian Hospital, Changhua 500209, Taiwan; 182919@cch.org.tw (J.-K.C.); 182985@cch.org.tw (C.-B.L.); 2 Big Data and Digital Promotion Center, Changhua Christian Hospital, Changhua 500209, Taiwan; 83686@cch.org.tw; 3 Doctoral Program in Translational Medicine, National Chung Hsing University, Taichung 402202, Taiwan; 4 Rong Hsing Translational Medicine Research Center, National Chung Hsing University, Taichung 402202, Taiwan

**Keywords:** intraoperative anastomotic leakage, Retzius-sparing, robot-assisted radical prostatectomy, postoperative urinary incontinence

## Abstract

*Background and Objectives*: The limited literature on the significance and risk factors of intraoperative anastomotic leakage (IAL) following Retzius-sparing robot-assisted radical prostatectomy (Rs-RARP) highlights the need for further investigation. This study aimed to assess the incidence of IAL, identify its associated risk factors, and evaluate its clinical implications. *Materials and Methods*: Patients with prostate adenocarcinoma who underwent Rs-RARP performed by a single surgeon between February 2015 and August 2023 were included in this study. Positive IAL was defined as the presence of anastomotic leakage identified through a water injection test performed immediately after vesicourethral anastomosis (VUA). Postoperative urinary continence was defined as the use of no pads or only a safety pad. Patients were categorized into two groups: those with positive IAL and those without. Immediate repair was performed in cases of positive IAL, and cystography was conducted approximately 10–14 days postoperatively. Chi-square test, Fisher’s exact test, Mann–Whitney U test, as well as univariable and multivariable logistic regression analyses, were used to evaluate the risk factors associated with IAL. Additionally, we analyzed the continence rate and the time to achieve continence following surgery. *Results*: A total of 230 patients underwent Rs-RARP for prostate adenocarcinoma performed by a single surgeon at our center during the aforementioned period. A water injection test was performed in all patients immediately after the VUA. IAL was observed in 32 patients (14%) during the water injection test. Postoperative cystography revealed very mild contrast medium leakage in only two patients (0.9%), with no impact on clinical recovery. No major IAL cases were identified on postoperative cystography. Patients with positive IAL required a significantly longer time to achieve continence compared to those without IAL (2.13 vs. 0.46 months, *p* = 0.008). Univariable analysis showed that a locally advanced T stage (>T2), longer console time, and absence of nerve-sparing were significantly associated with positive IAL. In multivariable analysis, a longer console time and a locally advanced T stage remained significant predictors of IAL. *Conclusions*: IAL detected by the water injection test was associated with the development of postoperative urinary incontinence and delayed recovery of continence. A tumor stage greater than T2 and longer console time were significant predictors of IAL. Further prospective randomized studies with larger sample sizes are required to validate our findings.

## 1. Introduction

Prostate cancer is the most frequently diagnosed malignancy and the second leading cause of cancer-related death among men in Western countries [1,2]. In Taiwan, it ranks as the fifth most common male cancer, with a steadily rising incidence over recent decades [3]. Data from the Taiwan Cancer Registry indicate that the age-adjusted incidence increased from 8.50 per 100,000 person-years in 1996 to 35.31 per 100,000 person-years in 2021 [4]. Radical prostatectomy remains the standard therapeutic option for localized prostate cancer, and the adoption of robot-assisted radical prostatectomy (RARP) has grown rapidly in recent years [5]. Despite the technical advancements of RARP, postoperative urinary incontinence remains a common and distressing complication that significantly affects patients’ quality of life and continues to be an unresolved challenge [6,7].

In 2010, Bocciardi et al. introduced the Retzius-sparing robot-assisted radical prostatectomy (Rs-RARP), a novel technique utilizing a retrovesical approach [8]. The procedure begins with incision of the peritoneum over the vas deferens and seminal vesicles, followed by careful dissection [8]. The prostate pedicle is identified by elevating the seminal vesicle, and the bladder neck is accessed via anterolateral dissection [8]. Depending on tumor characteristics, the neurovascular bundles are preserved or excised, and the bladder neck is incised posteriorly then anteriorly [8]. The prostate is mobilized and removed after urethral transection [8]. Pelvic lymph node dissection is performed when indicated, and urethrovesical anastomosis is completed using a running absorbable suture [8]. Rs-RARP preserves the Retzius space, maintaining key pelvic structures and promoting better early urinary continence and anatomical alignment [8]. However, the approach is technically challenging due to limited working space, reversed anatomy, and reduced access for lymphadenectomy, requiring experienced surgeons and careful patient selection [8]. Rs-RARP has demonstrated encouraging outcomes in promoting early return of urinary continence [8]. The precision and dexterity offered by robotic instrumentation, along with improved visualization of pelvic anatomy, have enabled more accurate and water-tight VUA during RARP [9]. Several studies have indicated that the quality of the VUA is closely linked to the surgeon’s experience, suggesting its utility as a marker of surgical expertise [10]. Compared to open retropubic radical prostatectomy, RARP has been associated with a lower incidence of vesicourethral anastomotic leakage, likely due to the improved quality of VUA [11].

Identifying the characteristics associated with intraoperative anastomotic leakage (IAL) is of clinical importance, as the presence of IAL has been correlated with delayed recovery of urinary continence [12]. Understanding the relevant risk factors can provide surgeons with meaningful insights to enhance operative technique and patient outcomes [5]. To date, only a limited number of studies have identified independent predictors of IAL through multivariable analysis in patients undergoing RARP [13]. Key factors reported include the absence of nerve-sparing, advanced patient age, larger prostate volume, shorter membranous urethral length, increased distance from the pubic bone to anterior rectum, and limited surgeon experience [14,15]. These variables have been shown to influence the occurrence of IAL and consequently affect postoperative continence recovery [14,15]. Therefore, awareness and recognition of these factors may assist surgeons in preoperative planning and intraoperative decision-making. To the best of our knowledge, this is the first study to investigate the clinical implications of IAL specifically during Rs-RARP, along with its associated risk factors.

## 2. Methods

### 2.1. Patient Population

This study was approved by the Institutional Review Board of Changhua Christian Hospital (IRB ID: 231021). All clinical data were prospectively collected in a customized database and retrospectively analyzed. Inclusion criteria: From February 2015 to August 2023, a total of 230 patients with prostate cancer underwent Rs-RARP via a transperitoneal approach. These procedures were performed by a single experienced surgeon using the Da Vinci Si Robotic System (Intuitive Surgical, Sunnyvale, CA, USA). Exclusion criteria are as follows: Patients were excluded if they had missing data or were lost to follow-up.

### 2.2. Surgical Technique

The surgical procedure followed the core principles established by Bocciardi and Galfano et al., and was consistently performed by a single surgeon [16]. VUA was carried out using a continuous running suture with two 3-0 V-Loc^™^ barbed sutures (Medtronic, Mansfield, MA, USA). A 20-Fr silicone Foley catheter (BARD, Covington, GA, USA)was inserted into the bladder, with the balloon inflated using 10 mL of distilled water. The anastomotic integrity was routinely assessed intraoperatively via a water-tightness test, in which 200–240 mL of normal saline was flushed through the Foley catheter. Positive IAL was defined as any observed leakage during this test, performed immediately after completion of the VUA. Whenever IAL was identified, immediate suture reinforcement was performed. We repaired leakage site with 3-0 V-Loc^™^ by single suture.

### 2.3. Postoperative Evaluation

For patients without IAL, the Foley catheter was removed between postoperative day 5 and 7. In contrast, those with confirmed IAL underwent cystography 10 to 14 days postoperatively to assess anastomotic healing, after which the catheter was removed. Continence was defined as the use of no pads or only a safety pad. The time to regain urinary continence was calculated from the date of surgery.

### 2.4. Statistical Analysis

Statistical analyses were conducted using SPSS version 22.0 (IBM Corp., Armonk, NY, USA). Patient and perioperative variables included age, body mass index (BMI), preoperative prostate-specific antigen (PSA) level, estimated blood loss, nerve-sparing status, prostate volume, console time, time interval since prostate biopsy, diabetes mellitus, hypertension, clinical T stage, and pathological features (Gleason score ≥ 8, pathological stage ≥ pT3). Continuous variables are presented as medians and interquartile ranges (25–75 percentile), while categorical variables are presented as numbers and percentages. The Mann–Whitney U test was used to compare median values between the two groups, while univariable analysis of categorical variables was performed using Fisher’s exact or Chi-square tests. Kaplan–Meier analysis was used to evaluate the time to urinary continence recovery, and comparisons were made using the log-rank test. Both univariable and multivariable logistic regression analyses were employed to identify factors independently associated with IAL. A two-sided *p*-value of < 0.05 was considered statistically significant. We also used ChatGPT 4o for English grammar correction.

## 3. Results

Patient demographics, baseline characteristics, and perioperative parameters are detailed in Table 1. To evaluate the integrity of the VUA intraoperatively, a standardized water injection test was systematically performed in every case immediately following the completion of the VUA. Based on the results of the water injection test, 198 patients (86%) were found to have a negative IAL, indicating a watertight VUA, while 32 patients (14%) exhibited positive IAL, defined by the presence of visible leakage at the anastomotic site. This rate aligns with previous reports in the literature and reflects the potential challenges associated with anastomotic precision, even in experienced hands. All patients with positive IAL received immediate intraoperative repair to reinforce the VUA, ensuring optimal watertightness. Postoperative evaluation included cystography performed between postoperative days 10 and 14 to assess for residual leakage before catheter removal. Mild contrast extravasation, defined as an area extending less than 4 cm^2^ from the leak point on cystography, was observed in only two patients (0.9%), both of whom had initially presented with positive IAL. Importantly, these minor leaks resolved spontaneously with continued catheter drainage and had no adverse impact on the overall recovery, continence outcomes, or length of hospital stay. No cases required reoperation or prolonged catheterization beyond the standard postoperative protocol.

A comparison between the negative and positive IAL groups demonstrated significant differences in several clinical and pathological parameters. Patients in the positive IAL group exhibited higher preoperative PSA levels and PSA density, suggesting a greater tumor burden prior to surgery. Moreover, these patients were more likely to present with a more advanced clinical stage and a higher proportion of high-risk disease. Intraoperatively, a significantly longer operative time was noted in the positive IAL group, which may reflect technical challenges associated with anastomotic integrity in patients with more complex disease. Pathological findings further supported the aggressiveness of disease in the positive IAL group, as evidenced by a higher pathological stage and greater tumor volume on final histopathology. The duration of Foley catheter placement post operation was significantly longer in the IAL positive group compared to the negative group. In contrast, no statistically significant differences were found between the two groups with respect to baseline characteristics such as BMI, the presence of diabetes mellitus, prostate volume, or the interval between prostate biopsy and surgery, indicating that general patient comorbidities and prostate anatomy were not major contributors to IAL occurrence. Additionally, estimated intraoperative blood loss was similar between groups, suggesting that the extent of surgical bleeding did not influence the risk of anastomotic leakage (Table 2).

**Figure 1:**
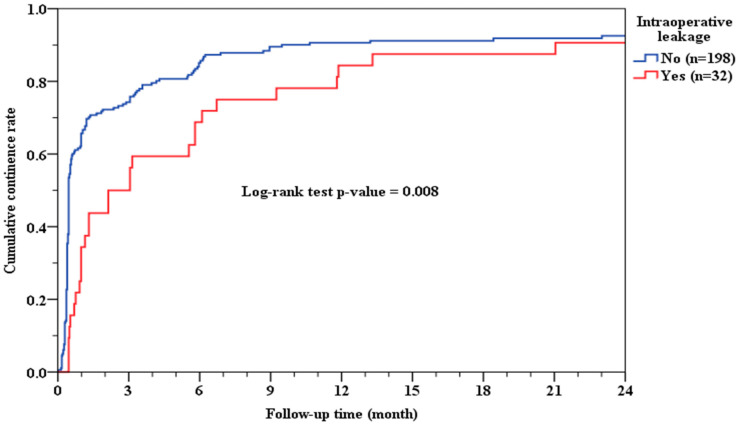
Cumulative continence rate and IAL.

Patients in the positive IAL group required a significantly longer duration to regain urinary continence compared to those without IAL. The median time to achieve continence was 2.13 months in the positive IAL group, whereas it was only 0.46 months in the negative IAL group (*p* = 0.008; Figure 1). This statistically significant delay suggests that the integrity of the VUA during surgery plays a crucial role in early postoperative functional recovery.

Univariable analysis revealed that several clinical and perioperative variables were significantly associated with the occurrence of IAL. Specifically, patients with longer console time, absence of nerve-sparing procedures, more advanced clinical T stage, and higher pathological grade group demonstrated a significantly greater likelihood of developing IAL (*p* = 0.002, 0.004, 0.001, and 0.031, respectively). To further investigate independent risk factors, variables with statistical significance in univariable analysis were included in a multivariable logistic regression model. The results showed that advanced clinical T stage (*p* = 0.023) and prolonged console time (*p* = 0.036) remained significant predictors of positive IAL. This indicates that tumor burden and procedural complexity are likely to affect the integrity of VUA, and may contribute to intraoperative leakage despite surgeon experience. The absence of nerve-sparing and higher grade group, although significant in univariable analysis, did not retain independent predictive value in the multivariable model (Table 3). There was no statistically significant association between surgeon experience and the occurrence of IAL, as determined by comparing the proportion of positive IAL cases across different years of surgical practice (Table 4). Although a slight variation in the annual incidence of IAL was observed, no consistent trend indicating improvement or deterioration over time was identified. This suggests that within the setting of an experienced high-volume surgeon, other factors—such as tumor characteristics and procedural complexity—may play a more decisive role in the development of IAL than chronological surgical experience alone. These findings imply that while accumulated experience contributes to overall surgical proficiency, it may not singularly influence specific technical outcomes such as anastomotic leakage during Rs-RARP.

## 4. Discussion

Recent studies have indicated that multiple factors should be considered when evaluating the occurrence of IAL in RARP [17]. These include patient-related variables, technical aspects of the surgery, and the surgeon’s experience [17]. A comprehensive analysis of these factors can offer valuable insights into the underlying causes of anastomotic leakage and inform strategies to prevent this complication, particularly in the context of Rs-RARP [17]. Kakutani et al. and Yanagida et al. previously reported a significant correlation between major IAL and prolonged time to achieve urinary continence following RARP [18]. A comparative study showed a significant improvement in immediate urinary continence with the Rs-RARP compared to the standard RARP technique, without a corresponding benefit in early potency recovery and with no increase in perioperative complications or positive surgical margin rates [19]. Rs-RARP also demonstrated improved urinary continence outcomes and reduced console time compared to conventional RARP, while maintaining similar complication rates [20]. Our findings are consistent with these reports, as we also observed that patients with positive IAL experienced delayed recovery of urinary continence after Rs-RARP. This statistically significant delay suggests that the integrity of the VUA during surgery plays a crucial role in early postoperative functional recovery. The presence of IAL may indicate technical stress or tissue fragility at the anastomotic site, potentially leading to local inflammation, delayed healing, or patient discomfort, all of which could contribute to prolonged incontinence. These findings underscore the importance of achieving a tension-free, watertight VUA intraoperatively to optimize early functional outcomes following Rs-RARP. Furthermore, these findings support the use of the intraoperative water injection test not only as a quality control measure but also as a potential prognostic indicator for postoperative urinary continence recovery. Anastomotic leakage may contribute to impaired healing at the vesicourethral junction, thereby affecting early functional outcomes.

In our multivariable analysis, both advanced clinical T stage and prolonged console time emerged as independent predictors of positive IAL. This indicates that tumor burden and procedural complexity are likely to affect the integrity of VUA, and may contribute to intraoperative leakage despite surgeon experience. This highlights the importance of early cancer detection and appropriate patient selection to reduce the risk of anastomotic complications. Additionally, longer console time may reflect increased technical complexity or intraoperative challenges, which could predispose to leakage at the VUA site. To ensure the reliability of VUA in prolonged console time or higher T stage patients, good surgical planning should be carried out [21]. By confirming tumor distribution and stage based on biopsy results and MRI images, the nerve-sparing region could be accurately identified, allowing for more efficient dissection [22]. As a result, reduced console time and minimal tissue damage could be observed. In addition, encouraging Kegel exercise training during the perioperative period can reduce the incidence of postoperative incontinence [23]. BMI has also been implicated as a risk factor for major anastomotic leakage [24,25]. Kakutani et al. identified higher BMI as a significant predictor, and Tung et al. further reported that obesity was associated with prolonged operative time and increased blood loss in extraperitoneal RARP [24,25]. However, they noted that functional outcomes were not negatively impacted by obesity [24,25]. In our study, BMI did not differ significantly between patients with and without IAL, suggesting that while obesity may increase surgical difficulty, it may not directly influence the incidence of leakage in a transperitoneal Rs-RARP setting. The reported incidence of anastomotic leakage varies widely in the literature, ranging from 0.3% to 15.4% according to Tyritzis et al. [26]. In our study, postoperative cystography was performed selectively for patients with intraoperative leakage, which may have led to an underestimation of the true leakage rate. Only two patients (0.8%) were found to have mild leakage on cystography, both of whom recovered without complications.

We also examined the interval between prostate biopsy and Rs-RARP as a potential factor influencing IAL. According to Lai et al., RARP can be safely performed at varying intervals after biopsy without compromising functional or oncological outcomes [27]. Our findings are in agreement with this, as no significant differences in IAL incidence were observed based on the timing of surgery following biopsy. Collectively, these findings provide meaningful insight into the risk factors and consequences associated with IAL. Understanding these associations is crucial for refining patient selection, enhancing intraoperative technique, and improving postoperative outcomes. Many studies have shown that nerve-sparing is closely associated with the recovery of urinary continence after surgery. Catarin et al. demonstrated that diminished sensitivity of the membranous urethra was correlated with urinary incontinence, particularly in individuals experiencing intermittent leakage. The continence mechanism is closely related to afferent autonomic innervation [28]. In addition, another study reported that the microcirculation of the membranous urethra was disrupted during radical prostatectomy [29]. In patients undergoing nerve-sparing radical prostatectomy, preservation of the neurovascular bundle may help maintain the vascular integrity of the membranous urethra. Another factor influencing urinary continence recovery after RARP is the preoperative evaluation of pelvic floor muscle thickness. Sho Hashimoto et al. measured the maximal diameters of the obturator internus and pubococcygeal muscles using MRI or computed tomography, and found that the diameters of these two muscles may help predict early postoperative urinary continence recovery in patients undergoing RARP [30].

This study has several limitations that should be acknowledged. First, it was conducted at a single tertiary medical center, and all procedures were performed by a single experienced surgeon using a consistent Retzius-sparing technique. While this ensured procedural standardization, it may limit the generalizability of our findings to other institutions, surgeons with different experience levels, or alternative surgical approaches. Second, although clinical data were prospectively collected, the analysis was retrospective in nature. This design inherently carries the risk of selection bias and limits the ability to establish causal relationships between risk factors and outcomes. Third, postoperative cystography was selectively performed only in patients with positive intraoperative leakage during the water injection test. As such, cases with subclinical or delayed leakage in the negative IAL group could have been missed, potentially leading to an underestimation of the true incidence of anastomotic leakage. Fourth, certain anatomical factors previously reported to influence anastomotic leakage, such as membranous urethral length or pubic symphysis–rectum distance, were not evaluated in this study due to limitations in imaging data. Lastly, the sample size was moderate, with 230 patients included over an 8.5-year period. Although this was sufficient for initial statistical analysis, a larger, multicenter cohort would provide greater power to detect subtle associations and validate the identified predictors. Future prospective, randomized studies with standardized postoperative imaging protocols and more diverse surgical settings are warranted to confirm and expand upon these findings.

## 5. Conclusions

IAL, as detected by the intraoperative water injection test, was significantly associated with delayed recovery of urinary continence following Rs-RARP. Advanced clinical T stage and prolonged console time were identified as independent predictors of IAL. These findings underscore the importance of meticulous surgical technique and appropriate case selection. Further validation through prospective randomized trials with larger sample sizes is necessary to confirm these results and guide future clinical practice.

## Figures and Tables

**Table 1:** Baseline characteristics of patients who underwent Rs-RARP.

Total (*n* = 230)			
	Characteristic	Median	(IQR)
Pre-op	Age (years)	67	(63–72)
	Height (cm)	165.5	(161.5–170.0)
	Weight (kg)	68	(60.4–75.0)
	BMI (kg/m^2^)	24.7	(22.8–27.0)
	DM (*n*/%)	45	(19.6)
	Pre-op PSA (ng/mL)	12.2	(7.8–25.5)
	Prostate volume (mL)	33	(26.4–42.2)
	PSA density	0.4	(0.2–1.0)
	Time to operation after biopsy (days)	56	(48–67)
	cT stage ≥ 3 (*n*/%)	67	(29.1)
	High risk (*n*/%)	113	(49.1)
Intra-op	Console time (min)	254	(217–288)
	Blood loss (mL)	150	(100–200)
	Without nerve-sparing (*n*/%)	64	(27.8)
	Pelvic lymph nodes dissection (*n*/%)	201	(87.4)
	Positive IAL (*n*/%)	32	(13.9)
	Negative IAL (*n*/%)	198	(86.1)
Post-op	pT stage ≥ 3 (*n*/%)	111	(48.7)
	Pathology Gleason ≥ 8 (*n*/%)	32	(14)
	Tumor volume (%)	20	(10–45)
	PSM (*n*/%)	128	(56)
	Hospitalization (days)	8	(7–9)
	Cystography (*n*/%)	32	(13.9)
	Overall leakage in cystography (*n*/%)	2	(0.8)

IQR: interquartile range; pre-op: preoperative; intra-op: intraoperative; post-op: postoperative; PSM: positive surgical margin; BMI: body mass index; DM: diabetes mellitus; PSA: prostate-specific antigen; IAL: intraoperative anastomotic leakage; high-risk disease: according to the D’Amico risk classification for prostate cancer.

**Table 2:** The comparison between negative IAL and positive IAL groups.

		Negative IAL (*n* = 198)		Positive IAL (*n* = 32)		*p*-Value
	Characteristic	Median	(IQR)	Median	(IQR)	
Pre-op	Age (years)	67	(63–72)	68	(65–74)	0.202
	Height (cm)	165.8	(161.5–170.0)	165.3	(161.6–169.0)	0.841
	Weight (kg)	68	(60.3–75.0)	67.5	(61.5–74.5)	0.992
	BMI (kg/m^2^)	24.8	(22.7–27.0)	24.5	(23.0–27.1)	0.96
	DM (*n*/%)	40	(20.2)	5	(15.6)	0.545
	* Pre-op PSA (ng/mL)	11.4	(7.0–24.8)	20.5	(11.9–43.1)	0.002
	Prostate volume (mL)	32.8	(26.3–41.3)	36.8	(28.9–51.0)	0.175
	* PSA density	0.4	(0.2–0.9)	0.5	(0.3–1.3)	0.036
	Time to operation after biopsy (days)	56	(48–67)	56	(42–62)	0.633
	** cT stage ≥ 3 (*n*/%)	49	(24.7)	18	(56.3)	<0.001
	* High risk (*n*/%)	89	(44.9)	24	(75)	0.002
Intra-op	** Console time (min)	249.5	(214–282)	277.5	(255–322)	<0.001
	Blood loss (mL)	150	(100–200)	150	(100–200)	0.67
	* Without nerve-sparing(*n*/%)	48	(24.8)	16	(50)	0.003
Post-op	* pT stage ≥ 3 (*n*/%)	88	(44.9)	23	(71.9)	0.004
	Pathology Gleason ≥ 8 (*n*/%)	24	(12.1)	8	(25)	0.098
	* Tumor volume (%)	20	(10–40)	37	(15–70)	0.049
	PSM (*n*/%)	106	(54)	22	(69)	0.121
	** Duration of catheter placement (days)	6	(5.0–7.0)	13	(7.5–17.0)	<0.001

*p*-value by Mann–Whitney U test or Chi-square test or Fisher’s exact test. Statistical significance is shown as * for *p* < 0.05, ** *p* < 0.01. IQR: interquartile range; pre-op: preoperative; intra-op: intraoperative; post-op: postoperative; PSM: positive surgical margin; BMI: body mass index; DM: diabetes mellitus; PSA: prostate-specific antigen; IAL: intraoperative anastomotic leakage; high-risk disease: according to the D’Amico risk classification for prostate cancer.

**Table 3:** Logistic regression analyses (univariable and multivariable) of IAL.

		Univariable Analysis (Crude)			Multivariable Analysis (Adjusted)		
Parameter		Odds Ratio	95% C.I.	*p*-Value	Odds Ratio	95% C.I.	*p*-Value
Pre-op PSA		1.008	0.998–1.018	0.099			
PSA density		1.161	0.843–1.599	0.360			
* Console time (mins)		1.009	1.003–1.015	0.002 **	1.007	1.000–1.013	0.036 *
* Nerve-sparing	Yes vs. No	0.320	0.149–0.688	0.004 **			
* Clinical T		2.322	1.385–3.891	0.001 **	1.896	1.094–3.286	0.023 *
* Grade group-pathology		1.391	1.031–1.877	0.031 *			

Statistical significance is shown as * for *p* < 0.05 and ** for *p* < 0.01.

**Table 4:** Positive IAL cases across different years.

Intraoperative Leakage (*n* = 32)					*p*-Value
OP year		Total	*n*	%
	Total	230	32	13.9
	2015	14	2	14.3	0.244
	2016	20	3	15.0	
	2017	21	1	4.8	
	2018	33	5	15.2	
	2019	34	10	29.4	
	2020	35	3	8.6	
	2021	20	4	20.0	
	2022	25	3	12.0	
	2023	25	1	3.5	

## Data Availability

The original contributions presented in the study are included in the article, and further inquiries can be directed to the corresponding authors.
